# Comparative effects of Gamal (*Gliricidia sepium*)-based diets supplemented with Gambier (*Uncaria gambir*) or direct-fed microbials on rumen fermentation, nutrient digestibility, microbial protein synthesis, and methane mitigation in beef cattle: An *in vitro* evaluation

**DOI:** 10.14202/vetworld.2025.3855-3869

**Published:** 2025-12-13

**Authors:** Bella Veliana Utami, Mardiati Zain, Elihasridas Elihasridas, Windu Negara, Laras Sukma Sucitra, Zaitul Ikhlas

**Affiliations:** 1 Doctoral Program, Faculty of Animal Science, Andalas University, Padang, West Sumatra, 25163, Indonesia; 2 Department of Animal Nutrition, Faculty of Animal Science, Andalas University, Limau Manis Campus, Padang, West Sumatra, 25163, Indonesia; 3 Research Center for Animal Husbandry, National Research and Innovation Agency, Jl. Raya Jakarta Bogor, Cibinong, 16915, Indonesia

**Keywords:** Gamal, methane reduction, rumen fermentation, direct-fed microbial, Gambier, digestibility

## Abstract

**Background and Aim:**

Enteric methane (CH_4_) emissions from ruminants reduce dietary energy efficiency and contribute to global greenhouse gas accumulation. *Gliricidia sepium (*Gamal), a protein-rich tropical legume, is widely used as a basal forage but may require targeted additives to optimize rumen fermentation. Plant bioactive compounds, such as *Uncaria gambir (*Gambier), and microbial supplements, such as direct-fed microbials (DFM) containing *Saccharomyces cerevisiae*, are known to influence fermentation pathways and energy use. This study aimed to compare the effects of Gamal-based diets supplemented with Gambier or DFMs on nutrient digestibility, rumen fermentation characteristics, volatile fatty acids (VFAs), microbial protein synthesis (MPS), fatty acid (FA) profiles, and CH_4_ mitigation under *in vitro* conditions.

**Materials and Methods:**

Six dietary treatments were evaluated in a randomized block design with three replicates: control, Gamal substitution, Gamal with 0.5% DFM, Gamal with 1% DFM, Gamal with 1% Gambier, and Gamal with 2% Gambier. Substrates were incubated in rumen fluid and buffer for 48 h at 39°C. Dry matter (DM) digestibility, organic matter digestibility, ammonia-nitrogen (NH_3_-N), VFAs, CH_4_ production, MPS, and FA composition were analyzed using standard procedures. Statistical analysis was performed using analysis of variance followed by Duncan’s test.

**Results:**

Supplementation with DFM improved DM and organic matter digestibility, with the highest values observed in the diet containing 0.5% DFM. The greatest methane reduction was observed with 1% Gambier, which lowered CH_4_ output by 33.43% compared with the control. DFM increased total VFAs and propionate concentration, reducing the acetate-to-propionate ratio. Gambier increased the acetate concentration and NH_3_-N and achieved the highest MPS values. FA profiles shifted according to additive type, with notable changes in saturated, monounsaturated, and polyunsaturated FA.

**Conclusion:**

Gamal-based diets supplemented with Gambier or DFM positively modified rumen fermentation, but through distinct mechanisms. DFM improved digestibility and fermentation stability, whereas Gambier achieved the greatest CH_4_ mitigation at 1% inclusion. Gambier represents a promising, locally available option for sustainable methane reduction in tropical ruminant feeding systems, supporting future in vivo validation.

## INTRODUCTION

Methane (CH_4_) is a potent greenhouse gas (GHG) and the second most significant contributor to global warming after carbon dioxide (CO_2_) [1]. Among agricultural sources, enteric fermentation in ruminants is the primary contributor, accounting for approximately 39% of total GHG emissions from the livestock sector [2]. This biological process not only exacerbates environmental burdens but also results in a 2%–12% loss of gross energy intake from feed, thereby reducing feed efficiency and overall animal productivity [3]. Consequently, reducing CH_4_ emissions from ruminants is crucial to improving nutrient utilization and promoting sustainable livestock production.

In Indonesia, beef cattle productivity remains below that of neighboring Southeast Asian countries, largely due to high feed costs, which can reach up to 70% of total production expenses [4, 5]. To enhance efficiency, the use of locally available, cost-effective feed additives is imperative. *Gliricidia sepium* (Gamal), a leguminous forage rich in protein, provides a sustainable feed resource but may elevate CH_4_ production if not properly balanced with fermentation-modulating additives. Plant-derived secondary metabolites, such as tannins and catechins, have been shown to inhibit methanogenesis by suppressing methanogenic archaea and reducing hydrogen (H_2_) availability [6]. Similarly, probiotics such as direct-fed microbials (DFMs) containing *Saccharomyces cerevisiae* are known to improve rumen fermentation, stabilize pH, and enhance fiber digestibility.

*Uncaria gambir* (Gambier), a plant native to West Sumatra, is rich in condensed tannins and catechins and represents a promising, locally sourced additive for CH_4_ mitigation. Gambier is positioned as a sustainable strategy for improving ruminant productivity while minimizing environmental impact, due to its dual potential to modulate rumen microbial activity and reduce enteric CH_4_ emissions [7].

Despite growing interest in sustainable feed additives for CH_4_ mitigation, research on the comparative effects of plant-based bioactive compounds and microbial probiotics in tropical livestock systems remains limited. Most existing studies have evaluated tannin-rich plants or microbial supplements separately, making it difficult to determine their relative effectiveness under similar experimental conditions. In Indonesia, Gamal and Gambier are locally abundant and cost-effective resources, yet their potential integration into ruminant diets to reduce CH_4_ emissions has not been fully explored. While Gambier is rich in condensed tannins and catechins known to suppress methanogenic archaea, its influence on rumen fermentation dynamics, nutrient digestibility, and microbial protein synthesis (MPS) has not been systematically compared with that of DFMs such as *S. cerevisiae*. Moreover, most available studies have been conducted under temperate conditions, leaving a gap in understanding how these additives perform in tropical feeding systems where high-fiber diets and environmental factors influence microbial fermentation. Addressing this gap is essential to developing region-specific, affordable strategies to improve feed efficiency and reduce CH_4_ emissions in smallholder beef cattle production systems.

This study aimed to compare the effects of Gambier and direct-fed microbial supplementation using *S. cerevisiae* on rumen fermentation characteristics, nutrient digestibility, MPS, fatty acid (FA) composition, and CH_4_ production in an *in vitro* fermentation system based on a Gamal–containing beef cattle diet. The study further sought to evaluate how these two additives differentially modulate microbial activity and fermentation pathways in Gamal-based substrates, and to determine the optimal inclusion levels of Gambier and DFM for enhancing fermentation efficiency and reducing CH_4_ emissions under controlled *in vitro* conditions.

## MATERIALS AND METHODS

### Ethical approval

This *in vitro* study did not involve live animals and therefore did not require formal ethical approval. However, all procedures related to the collection and handling of rumen fluid were conducted in accordance with the Animal Ethics Committee of the Faculty of Animal Science, Andalas University. Rumen contents were obtained from cattle immediately after slaughter at an authorized municipal abattoir under the supervision of official veterinary officers, ensuring adherence to animal welfare and biosafety protocols.

### Study period and location

This study was conducted from October 2024 to January 2025 at the Laboratory of Ruminants, Faculty of Animal Science, Andalas University, and Laboratory Animal Logistics Indonesia–Netherlands, Bogor, Indonesia.

### Sample preparation

#### Forage collection and processing

*Pennisetum purpureum (*Napier grass) and Gamal were collected from the Faculty of Animal Science, Andalas University, UPT Teaching Farm at approximately 0°54’33.6”S, 100°27’53.2”E. According to the Köppen classification, the region has a tropical rainforest climate (Af), with an average temperature of 26°C–28°C and annual rainfall of around 3,000 mm. These conditions are typical of lowland tropical zones and influence the nutrient profile of forages used in the experiment. The chemical composition details are provided in Table 1. Napier grass was harvested at 50 days of age and cut into smaller pieces for drying. The forage was sun-dried for 1–2 days, depending on sunlight intensity, until the moisture content decreased. Subsequently, the drying process was continued in an oven at 60°C for 24 h until the forage was sufficiently dried for grinding. The dried samples were then ground using a grinder machine and sieved through a 1 mm mesh to obtain a uniform texture. The prepared samples were stored in plastic clip bags and refrigerated until analysis.

**Table 1:** Chemical composition of the feed material (% DM).

Nutritional composition (%)	Napier grass	Gamal	PKM	Rice bran	Corn	Mineral
DM	90.01	88.29	85.91	85.87	85.55	100
Organic matter	90.88	91.60	96.13	88.99	95.87	0.00
Ash	9.12	8.40	3.87	11.01	4.13	0.00
Crude protein	9.66	25.7	22.29	8.94	9.82	0.00
Crude fiber	32.6	13.30	29.96	15.62	9.61	0.00
Extract ether	2.06	1.97	10.99	8.76	3.09	0.00
NDF	65.16	26.41	72.59	48.11	58.62	0
ADF	41.21	21.78	50.33	23.08	34.66	0
NFE	46.56	50.63	32.89	55.67	73.35	0.00
TDN	55.00	60.39	76.85	68.98	77.69	0.00

DM = Dry matter, PKM = Palm kernel meal, NDF =Neutral detergent fiber, ADF = Acid detergent fiber, NFE = Nitrogen-free extract, TDN = Total digestible nutrient.

#### Concentrates and preparation of Gambier

Gambier was a commercial dried extract (brownish powder) purchased from local producers in West Sumatra, Indonesia. The extract contained 55.21% catechins and 60.22% total tannins as determined by spectrophotometric analysis (Shimadzu UV-1800, Shimadzu Corporation, Japan). Gambier was stored in airtight containers at room temperature (25°C–27°C) and protected from light until use. The powder was directly incorporated into the substrate mixture according to the treatment levels (1% and 2% of total dry matter [DM]). The concentrates used in this study included RBS, palm kernel meal, and corn. The chemical compositions of the concentrates are shown in Table 2. The DFM used in this study was commercial yeast (*S. cerevisiae*) obtained from a local baking supplier (Fermipan, PT Lesaffre Indonesia, Indonesia). DFM was included in the diet at levels of 0.5% and 1% (w/w).

**Table 2:** Composition and nutritional content of the 100% concentrate.

Concentrate composition	% DM
PKM	40
Rice bran	33
Corn	25
Minerals	2
Total	100
**Nutritional composition**	
Dry matter	86.09
Organic matter	91.79
Ash	6.21
ADF	36.41
NDF	59.57
Crude protein	14.32
Crude fiber	19.54
Crude fat	8.06
NFE	49.86
TDN	72.93

DM = Dry matter, PKM = Palm kernel meal, NDF =Neutral detergent fiber, ADF = Acid detergent fiber, NFE = Nitrogen-free extract, TDN = Total digestible nutrient.

### Experimental design and dietary treatments

The experimental design consisted of six treatments evaluated in a randomized block design, with each treatment replicated 3 times (n = 3). The use of three replicates is a standard practice in *in vitro* gas production studies [8, 9], which ensures sufficient reliability to capture biological variability, measure fermentation parameters, and detect statistically significant differences among treatments under controlled *in vitro* conditions. This design has also been widely applied in ruminant CH_4_ mitigation studies. Treatments are shown in Table 3. Thus, P0 was used as the negative control (no supplementation), while P1 (with Gliricidia) served as the basal substrate for comparison with either DFM or Gambier supplementation. The experimental design aimed to evaluate the effects of different levels of Gambier and DFM supplementation on the overall diet composition. Nutrients in the experimental diet are presented in Table 4.

**Table 3:** Dietary treatment composition used in the *in vitro* study.

Treatment	Composition (DM basis)	Description
P0	40% Napier grass + 60% Concentrate	Control
P1	40% Napier grass + 30% Concentrate + 30% Gamal	Control + Gamal (basal substarte)
P2	40% Napier grass + 30% Concentrate + 30% Gamal + 0.5% DFM	Basal + 0.5% *S. cerevisiae*
P3	40% Napier grass + 30% Concentrate + 30% Gamal + 1% DFM	Basal + 1% *S. cerevisiae*
P4	40% Napier grass + 30% Concentrate + 30% Gamal + 1% Gambier	Basal + 1% Gambier extract
P5	40% Napier grass + 30% Concentrate + 30% Gamal + 2% Gambier	Basal + 2% Gambier extract

DM = Dry Matter, DFM = Direct-fed microbial, *S. cerevisiae* = *Saccharomyces cerevisiae*

**Table 4:** Chemical composition of the experimental diets (% DM).

Feed composition and chemical compositions	Feed treatment	

	RK	RG
Napier grass	40	60
Concentrate	60	30
Gamal	0	30
DM	87.76	88.32
Organic matter	91.42	91.37
Ash	7.38	8.03
ADF	38.33	33.94
NDF	61.80	51.86
Crude protein	12.46	15.87
Crude fiber	24.76	22.89
Crude fat	5.66	3.83
NFE	48.54	48.77
TDN	65.76	61.99
Tannin	0.89	3.28

DM = Dry Matter, RK (Control feed = Napier grass 40% + Concentrate 60%); RG (Gamal feed = Napier grass 40% + Concentrate 30% + Gamal 30%), ADF = Acid detergent fiber, NDF =Neutral detergent fiber, NFE = Nitrogen free extract, TDN = Total digestible nutrient

### *In vitro* fermentation

#### Rumen fluid collection

The *in vitro* procedures used in this study were adapted from Tilley and Terry [8] to determine the digestibility and rumen fermentation characteristics. Rumen fluid was collected in the morning from animals that were slaughtered for human consumption at a local slaughterhouse. The ingesta was squeezed and transferred into a thermos, which was maintained at 39°C to ensure temperature stability and preserve the anaerobic environment for microbial activity. The fresh rumen liquor was filtered through a nylon sieve (100 µm) and then diluted with the McDougall buffer solution [10] at a ratio of 1:4 (rumen fluid:buffer solution).

#### Incubation procedure

Each 300 mL Erlenmeyer flask was anaerobically filled with 2.5 g of feed sample and 250 mL of mixed solution (rumen fluid and buffer) by pumping CO_2_ gas into the flask and sealing it with a rubber lid. Incubation was conducted in a shaker incubator at 39°C with a rotation speed of 90 rpm for 48 h. Fermentation was halted by immersing the Erlenmeyer flasks in ice water to stop microbial activity, and the pH was measured afterward. The next step involved separating the supernatant and residue by centrifuging the samples (Hitachi Koki Co., Japan) at 1,006 × *g* for 15 min. The supernatant was analyzed for rumen fermentation characteristics, and the residue was used to assess feed digestibility. The supernatant was transferred to bottles and stored in a freezer for analysis of ammonia (NH_3_), VFAs, MPS, and FA profile. The residue was then filtered using Whatman No. 41 filter paper (Cytiva, UK) and dried in an oven at 60°C for 48 h. Each treatment was incubated in triplicate within each run, and the entire experiment was repeated in triplicate.

### Analytical procedures

#### Digestibility

The *in vitro* DM digestibility (IVDMD) and *in vitro* organic matter digestibility (IVOMD) were determined following the proximate analysis method [11]. After incubation, the undigested residue was filtered through Whatman No. 41 filter paper, oven-dried at 60°C for 24 h, and weighed to calculate IVDMD and IVOMD. All measurements were performed in triplicate, and the results are expressed as mean ± standard deviation (SD).

#### Rumen fermentation parameters (pH, NH_3_, VFA, and CH_4_)

The pH of the fermentation medium was measured immediately after incubation using a digital pH meter (Eutech Instruments, Singapore, model pH 700).

NH_3_ nitrogen concentration was determined using Conway’s microdiffusion method [12]. One milliliter of the supernatant was pipetted into one compartment of a Conway’s dish, and 1 mL of sodium carbonate solution was placed in the opposite compartment. The center well contained 1 mL of boric acid as the trapping reagent. The dish was sealed with petroleum jelly and incubated at room temperature for 24 h to allow NH_3_ diffusion, followed by titration with 0.005 N sulfuric acid (H_2_SO_4_). The NH_3_ concentration was calculated based on the volume of acid used.

The partial VFA profile was determined using gas chromatography (GC) (Hewlett-Packard 5890, USA) equipped with a flame ionization detector (FID). A stainless-steel column (0.20 cm inner diameter, 0.40 cm outer diameter) was used for separation. Pure nitrogen served as the carrier gas at a flow rate of 0.5 mL/s, while H_2_ and oxygen were supplied at 0.5 and 5 mL/s, respectively, for combustion. The column temperature was maintained isothermally at 125°C, with the injector and detector temperatures set at 160°C and 200°C, respectively. A standard mixture of acetate, propionate, and butyrate (Supelco^®^, Merck KGaA, Darmstadt, Germany) was used for calibration. Individual VFA components were identified by comparing retention times with those of standard mixtures.

CH_4_ production was measured using the gas syringe technique developed by Fievez *et al*. [13]. CH_4_ is separated from other gases using a sodium hydroxide (NaOH) solution. CH_4_ was measured by passing the collected gas through 10 mL of NaOH solution (10 M), which absorbs CO_2_ and other acidic gases. The remaining gas is assumed to be CH_4_, and its volume is recorded. This CH_4_ value is then calculated based on 1 g of digested DM and expressed as g/mL of digested DM.

#### MPS

MPS was estimated using the Lowry method [14]. Absorbance was measured at 650 nm using an ultraviolet-visible spectrophotometer, and bovine serum albumin was used as the standard curve.

#### FA profile

FA analysis was performed through three main stages: fat extraction, methylation, and chromatographic separation using a Shimadzu GC-2014 (Shimadzu Manufacturing Inc., Canby, OR, USA). The procedure began by stopping the *in vitro* incubation by adding 500 μL of a 2% mercury (II) chloride solution (w/v). The contents of each incubation tube were then transferred into a 100 mL glass flask and stored at 60°C before being freeze-dried for over 48 h to ensure complete water removal. Approximately 50 mg of the freeze-dried sample was weighed into a screw-cap tube for methylation. The process was conducted by adding 2148 μL of methanol, 990 μL of toluene, 66 μL of 99.9% H_2_SO_4_, 1000 μL of dimethyl sulfoxide, and 2 mL of hexane. Subsequently, the mixture was incubated in a water bath at 80°C for 2 h. Once cooled to room temperature, the upper hexane layer was carefully collected into an Eppendorf tube. Subsequently, the solvent was evaporated under a nitrogen stream, and the residue was redissolved in 500 μL of dichloromethane. From this solution, 250 μL was injected into a Shimadzu GC-2014 equipped with a GC-FID and an autosampler. FA separation was achieved using a fused silica capillary column RT-2560, 100 m × 0.25 mm i.d., 0.20 µm film thickness; Restek, USA) with helium as the carrier gas flowing at 1.12 mL/min. FA identification was determined by comparing their retention times with those of the Supelco 37 Component FAME Mix standard. All analyses, including CH_4_, NH_3_, VFAs, and FA profiles, were performed in triplicate (n = 3) to ensure data reliability and reproducibility. The mean values and SDs were calculated from these replicates. The coefficient of variation across replicate analyses was maintained below 5%. The detection limit for individual FAs was 0.01 mg/mL.

### Statistical analysis

Data normality and homogeneity of variance were tested using Shapiro–Wilk and Levene’s tests, respectively. Results are presented as mean ± SD. Statistical analyses were performed using IBM Statistical Package for the Social Sciences Statistics version 29.0 (IBM Corp., Armonk, NY, USA) [15]. All data were processed and analyzed for variance using analysis of variance, followed by Duncan’s multiple range test.

## RESULTS

### Data expression and units

All results are expressed using consistent units: CH_4_ as milliliters per gram of digested DM (DDM; mL/g), NH_3_ nitrogen concentration as milligrams per deciliter (mg/dL), VFAs concentration as millimolar, and FAs as the percentage of total identified fatty acid methyl esters (% of total FAMEs).

### Nutrient digestibility

DMD and organic matter digestibility (OMD) were not significantly affected (p > 0.05) by either treatment, whether with the supplementation of Gambier or DFM. The observed range for DMD was 53.56%–63.62%, while the range for OMD was 57.62%–66.77%, as presented in Table 5.

**Table 5:** Nutrient digestibility.

Nutrient digestibility (%)	Treatments					

	P0	P1	P2	P3	P4	P5
DMD	53.56^c^ ± 1.14	56.09^b^± 0.57	63.62^a^± 1.32	62.22^a^± 1.09	62.05^a^ ± 0.96	54.18^c^ ± 0.79
OMD	57.62^c^± 0.84	59.89^b^± 0.41	66.77^a^± 1.19	65.49^a^± 0.56	65.34^a^± 0.85	58.15^c^± 0.25

^a–c^ Values in the same row with different superscript letters differ significantly (p < 0.05). P0 = 40% Napier grass + 60% Concentrate, P1 = 40% Napier grass + 30% Concentrate + 30% Gamal, P2 = 40% Napier grass + 30% Concentrate + 30% Gamal + 0.5% DFM, P3 = 40% Napier grass + 30% Concentrate + 30% Gamal + 1% DFM, P4 = 40% Napier grass + 30% Concentrate + 30% Gamal + 1% Gambier, P5 = 40% Napier grass + 30% Concentrate + 30% Gamal + 2% Gambier, DMD = Dry matter digestibility, OMD = Organic matter digestibility, DFM = Direct-fed microbial.

### Rumen fermentation characteristics

The ruminal fermentation characteristics of all treatments are summarized in Table 6. The rumen fermentation parameters were significantly affected by the treatments. The pH values remained relatively stable across all treatments, ranging from 6.72 to 6.90, with no significant differences (p > 0.05), indicating that fermentation conditions were maintained within the optimal range for microbial activity.

**Table 6:** Characteristics of rumen fermentation.

Characteristics of rumen fermentation	Treatments					

	P0	P1	P2	P3	P4	P5
pH	6.87 ± 0.06	6.81 ± 0.09	6.80 ± 0.01	6.81 ± 0.05	6.87 ± 0.03	6.76 ± 0.01
NH_3_ (mg/dL)	21.14^a^ ± 7.17	24.03^ab^ ± 9.42	24.88^b^ ± 10.65	24.82^b^ ± 9.39	25.11^b^ ± 6.69	25.39^b^ ± 7.77
Acetic acid (C2) (mM)	82.68^a^ ± 0.86	67.14^c^ ± 0.63	74.92^b^ ± 0.30	21.65^f^ ± 1.01	35.78^d^ ± 0.53	29.02^e^ ± 0.33
Propionic acid (C3) (mM)	28,35^b^ ± 0.62	44,95^a^ ± 0.15	23,45^c^ ± 0.48	17,68^d^ ± 0.09	10,18^e^ ± 0.07	11,98^f^ ± 0.07
Butyric acid (C4) (mM)	6.08^c^ ± 0.17	14.10^a^ ± 0.03	7.99^b^ ± 0.15	5.37^d^ ± 0.06	4.25^e^ ± 0.05	3.33^f^ ± 0.08
Valeric acid (C5) (mM)	1.16^c^ ± 0.14	2.18^a^ ± 0.18	1.42^b^ ± 0.03	1.10^c^ ± 0.02	0.71^d^ ± 0.04	0.74^d^ ± 0.09
Isobutyric acid (iC4) (mM)	1.56^b^ ± 0.06	2.16^a^ ± 0.04	1.22^c^ ± 0.15	1.31^c^ ± 0.02	2.03^a^ ± 0.03	1.21^c^ ± 0.07
Iso-valeric acid (iC5) (mM)	2.22^b^ ± 0.29	3.05^a^ ± 0.09	1.71^c^ ± 0.06	2.27^b^ ± 0.09	2.15^b^ ± 0.04	1.73^c^ ± 0.09
VFA total	122.05^b^ ± 0.50	133.58^a^ ± 0.70	110.72^c^ ± 1.15	49.37^e^ ± 1.18	55.10^d^ ± 0.49	48.01^e^ ± 0.67
Ratio A:P	2.92^c^ ± 0.08	1.49^e^ ± 0.01	3.20^b^ ± 0.05	1.22^f^ ± 0.05	3.52^a^ ± 0.06	2.42^d^ ± 0.02
Gas methane/digested DM (mL/g)	21.53^a^ ± 3.05	20.62^ab^ ± 0.44	17.28^abc^ ± 2.62	17.68^abc^ ± 4.43	14.33^c^ ± 0.89	14.60^bc^ ± 0.76
MPS (mg/100 mL)	61.85^e^ ± 5.48	102.55^d^ ± 9.30	146.06^c^ ± 2.28	162.98^b^ ± 2.40	197.73^a^ ± 3.67	207.70^a^ ± 9.08

Values in the same row with different superscript letters differ significantly (p < 0.05). P0 = 40% Napier grass + 60% Concentrate, P1 = 40% Napier grass + 30% Concentrate + 30% Gamal, P2 = 40% Napier grass + 30% Concentrate + 30% Gamal + 0.5% DFM, P3 = 40% Napier grass + 30% Concentrate + 30% Gamal + 1% DFM, P4 = 40% Napier grass + 30% Concentrate + 30% Gamal + 1% Gambier, and P5 = 40% Napier grass + 30% Concentrate + 30% Gamal + 2% Gambier, VFA = Volatile fatty acid, DM = Dry matter, MPS = Microbial protein synthesis.

NH_3_–nitrogen concentrations increased gradually with supplementation, with the highest level observed in the 2% Gambier treatment (25.39 mg/dL), which was significantly higher than the control and other treatments (p < 0.05). Overall, NH_3_ levels ranged from 21.14 to 25.39 mg/dL of rumen fluid, suggesting enhanced deamination activity in the presence of Gambier.

Distinct patterns were also observed in the VFA profiles, with significant differences in the concentrations of individual VFAs. Acetic acid (C_2_) levels varied considerably, with the control group exhibiting the highest concentration (82.68%) and the 1% DFM treatment showing the lowest (21.65%) (p < 0.05). The concentrations of propionic acid (C_3_) and butyric acid (C_4_) were highest in treatment P1, which involved the substitution of Gamal, with corresponding values of 44.95% and 14.10%, respectively. These values were significantly higher than those of the control and other treatments (p < 0.05).

The total VFA concentration was highest in P1, which differed significantly from the other treatments, ranging from 48.01 to 133.58 mM. The acetate-to-propionate (A:P) ratio was significantly higher in the 1% Gambier treatment (3.52) than in the P3 (1% DFM) and P1 treatments (1.22 and 1.49, respectively) (p < 0.05). These results demonstrate that both additives modulated fermentation patterns differently: Gambier favored acetate production, while DFM enhanced propionate formation.

#### CH_4_ gas production

**Figure 1:**
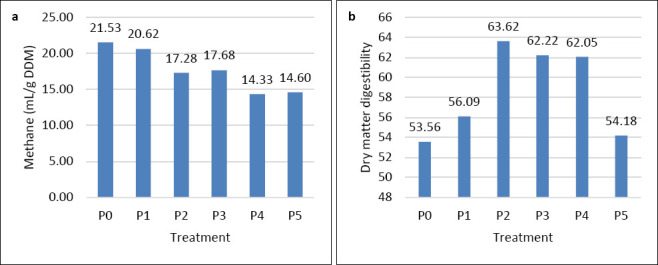
(a) Methane gas production. (b) Digestibility of dry matter.

CH_4_ yield varied significantly among treatments (Figures 1a and 1b). The control (P0) produced the highest CH_4_ yield (21.53 mL/g DDM), whereas treatment P4 (1% Gambier) produced the lowest (14.33 mL/g DDM). CH_4_ production was markedly reduced compared with the control (P0).

At 1% and 2% Gambier inclusion levels (P4 and P5), CH_4_ decreased by 33.43% and 32.17%, respectively. This reduction corresponds to approximately 6.7 L CH_4_/day/animal, or 1.34 kg CO_2_-equivalent/day at a smallholder scale (10 goats/farm), representing an annual mitigation potential of nearly 490 kg CO_2_-equivalent per farm.

Yeast supplementation at 0.5% (P2) and 1% (P3) also reduced CH_4_ emissions by 19.73% and 17.87%, respectively. These reductions demonstrate the strong potential of both additives to mitigate CH_4_ emissions during *in vitro* fermentation. The results are also illustrated graphically (Figures 1a and 1b) to enhance clarity, showing the trends in CH_4_ reduction and digestibility across all treatments.

### FA profile

The FA composition of the rumen fermentation products under different treatments is presented in Table 7. Palmitic acid (C16:0) was the predominant saturated FA (SFA) across all treatments, with the highest concentration observed in Treatment P3. Stearic acid (C18:0) and myristic acid (C14:0) were also major SFAs, although their concentrations remained relatively stable among treatments.

**Table 7:** Fatty acid composition of the treatment was analyzed by gas chromatography.

Fatty acid	Treatments					

	P0	P1	P2	P3	P4	P5
C14:0 (Myristic)	6.97 ± 4.89	6.86 ± 2.46	5.71 ± 1.95	6.57 ± 1.61	3.63 ± 1.70	5.97 ± 2.93
C16:0 (Palmitic)	28.07^b^ ± 9.44	27.51^b^ ± 10.82	29.59^ab^ ± 2.32	40.35^a^ ± 4.69	37.71^ab^ ± 0.94	38.75^ab^ ± 6.43
C18:0 (Stearic)	26.38 ± 6.25	25.65 ± 5.20	20.98 ± 7.15	20.35 ± 0.81	21.75 ± 5.11	20.43 ± 0.78
C18:1 n-9 (Oleic)	1.76^ab^ ± 0.54	1.15^ab^ ± 0.002	3.96^a^ ± 3.87	0.72^b^ ± 0.25	1.10^ab^ ± 0.49	0.92^ab^ ± 0.30
C18:2 n-6 (Linoleic)	0.90 ± 0.32	0.92 ± 0.26	0.60 ± 0.22	0.68 ± 0.11	0.66 ± 0.40	0.70 ± 0.04
C18:3 n-3 (alpha-linolenic)	0.40 ± 0.02	0.69 ± 0.23	0.47 ± 0.18	0.49 ± 0.10	0.59 ± 0.11	0.53 ± 0.05
C20:0 (Arachidic)	1.23^ab^ ± 0.48	1.27^ab^ ± 0.50	0.91^ab^ ± 0.31	1.12^ab^ ± 0.24	1.48^a^ ± 0.06	0.74^b^ ± 0.15
Total SFA	76.32 ± 1.37	77.22 ± 3.30	70.70 ± 9.07	80.17 ± 1.64	77.16 ± 5.22	78.38 ± 2.50
Total MUFA	13.99 ± 0.86	11.54 ± 0.68	16.50 ± 6.68	10.08 ± 1.05	13.30 ± 3.36	12.20 ± 2.06
Total PUFA	7.16 ± 0.47	9.27 ± 2.51	10.92 ± 3.22	7.23 ± 0.26	7.78 ± 2.35	7.44 ± 1.22
Total CLA	2.53 ± 0.09	1.96 ± 0.57	1.88 ± 0.39	2.52 ± 0.81	1.76 ± 0.93	1.98 ± 0.29

Values in the same row with different superscript letters differ significantly (p < 0.05). P0 = 40% Napier grass + 60% Concentrate, P1 = 40% Napier grass + 30% Concentrate + 30% Gamal, P2 = 40% Napier grass + 30% Concentrate + 30% Gamal + 0.5% DFM, P3 = 40% Napier grass + 30% Concentrate + 30% Gamal + 1% DFM, P4 = 40% Napier grass + 30% Concentrate + 30% Gamal + 1% Gambier, P5 = 40% Napier grass + 30% Concentrate + 30% Gamal + 2% Gambier, SFA = Saturated fatty acids, MUFA = Monounsaturated fatty acids, PUFA = Polyunsaturated fatty acids, CLA = Conjugated linoleic acid.

Among the monounsaturated FAs (MUFAs), oleic acid (C18:1 n-9) was most elevated in Treatment P2, while the lowest concentration was recorded in P3. The polyunsaturated FAs (PUFAs), particularly linoleic acid (C18:2 n-6) and α-linolenic acid (C18:3 n-3), showed minor variations, with the highest total PUFA content found in P2.

Conjugated linoleic acid (CLA) levels varied slightly among treatments, with P0 and P3 showing relatively higher concentrations. These findings indicate that the different feed supplementations had a measurable impact on the ruminal FA composition, particularly in modulating the proportions of SFA and MUFA.

### Summary of findings

Overall, both Gambier and DFM supplementation influenced rumen fermentation characteristics, gas production, and FA composition without significantly affecting nutrient digestibility. Gambier supplementation notably reduced CH_4_ emissions and increased NH_3_ concentrations, whereas DFM improved propionate production and maintained fermentation stability. These synergistic effects demonstrate the potential of both additives as environmentally sustainable feed supplements in ruminant nutrition.

## DISCUSSION

### Nutrient digestibility

This study on DMD revealed significant variation (p < 0.05) among the different feed treatments. The control treatment (P0) had the lowest DMD (53.56%), whereas treatments P2–P5 had higher values. Treatments P1, P2, and P3 demonstrated superior digestibility compared with the control. Treatments P2 and P3 recorded nearly identical DMD values (62.22% and 62.05%, respectively), suggesting that both the substitution of concentrate with leguminous plants, such as Gamal, and the inclusion of DFMs were effective in enhancing DMD. In contrast, treatments P4 and P5, which incorporated Gambier, yielded lower DMD values (54.18%), indicating that Gambier in feed formulations was less effective at improving digestibility than DFM-based treatments.

### Effect of leguminous inclusion

The inclusion of leguminous plants, such as Gamal, in livestock feed significantly enhances DMD. Legumes have higher protein content and are more readily fermentable by rumen microbes than grass- or concentrate-based feeds. This is attributed to the favorable nutritional profile and protein content of legume leaves, which support microbial fermentation and nutrient absorption in the rumen. A previous study by Paat *et al*. [16] has shown that adding legume leaves to silage improves both the nutritional value and digestibility of silage. Similarly, Sofyan *et al*. [17] reported that incorporating Gamal leaves into silage enhances the digestibility of dry and organic matter. Legumes such as Gamal supply rumen-degradable protein that promotes microbial growth, although antinutritional factors such as tannins and saponins present in Gamal may inhibit the absorption of certain nutrients, thereby influencing feed efficiency and overall DMD in ruminants.

### Effect of DFMs

Treatments supplemented with DFM, particularly P2 (0.5% DFM) and P3 (1% DFM), showed improved digestibility compared with the control. The highest DMD observed in this study was in P2 treatment. These findings differ slightly from those of Mathukiya [18], who reported that up to 3% DFM supplementation increased IVDMD to 57.03%. This supports the notion that DFM supplementation can enhance feed digestion and nutrient absorption. However, the effectiveness of DFM appears to be influenced by its interaction with other dietary components, particularly the types of forage and concentrate used. The impact of DFM on nutrient digestibility also depends on the microbial strains used, their dosage, and the animal’s specific dietary conditions [19, 20].

### Effect of Gambier supplementation

The feed treatments supplemented with Gambier showed an opposite trend: digestibility decreased as the dosage increased. The 1% Gambier supplementation (P4) resulted in a DMD value of 62.05%, which was still higher than that of P5. This suggests that higher levels of Gambier supplementation may negatively affect fermentation processes and DMD. This effect is likely due to the high tannin content in Gambier, which can bind to proteins and digestive enzymes, thereby inhibiting rumen microbial activity in the breakdown of feed components.

### OMD

**Figure 2:**
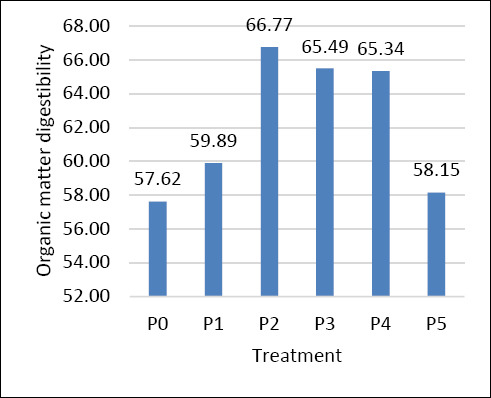
Digestibility of organic matter.

In this study, OMD also showed significant variation among treatments (Figure 2), indicating that the substitution of concentrate with Gamal and the addition of either DFMs or *Gambier* influenced feed digestibility. The highest OMD was observed in P2 (66.77%), followed by P3 (65.49%) and P4 (65.34%), whereas the lowest OMD was observed in P0 (57.62%) and P5 (58.15%).

The improved digestibility observed in P2 may be attributed to a synergistic effect between Gamal substitution and DFM supplementation. As a legume, Gamal contains relatively high crude protein and more digestible fiber than grasses, thereby enhancing rumen fermentation. Supplementation with 0.5% DFM likely enhanced rumen microbial activity, improved fiber degradation, and increased nutrient use. DFMs containing *S. cerevisiae* or lactic acid bacteria can stabilize rumen pH, enhance cellulolytic bacterial populations, and improve feed digestibility [21–23]. However, a higher DFM dosage, as in P3 (1%), did not further improve OMD, suggesting that 0.5% may be the optimal microbial supplementation level.

The control ration (P0) showed the lowest OMD, possibly due to a high concentrate proportion, which may have led to excessively rapid fermentation, suboptimal rumen pH, and reduced fiber digestion [24, 25]. Meanwhile, P5 (2% Gambier) exhibited low digestibility, likely due to the excessive tannin content in Gambier, which inhibits microbial enzymes and reduces nutrient availability. Jayanegara *et al*. [26] reported that increasing tannin levels significantly reduced the digestibility of crude protein and fiber, with a more pronounced effect on protein digestion. Although overall OMD may not decline significantly, the negative effects on specific nutrients can impair total nutrient use. High tannin concentrations can reduce both DMD and total short-chain FA production in ruminants. This study found that tannins, particularly hydrolyzable tannins, negatively affect rumen fermentation and nutrient availability [27].

### Rumen fermentation characteristics

#### Rumen pH

The average rumen pH values ranged from 6.72 to 6.90 (Table 5). These findings align with those of Silva *et al*. [28], who demonstrated that the inclusion of a DFM-A mixture resulted in a higher average ruminal pH (5.67) than in the control group (5.50). The increase in pH suggests an improved ruminal environment, potentially supporting more stable microbial activity and fermentation processes. The results of this study indicated that rumen pH remained within the optimal physiological range for cattle, generally considered to be 6.79–6.92 [29]. Maintaining this pH level is crucial to prevent rumen acidosis and ensure proper microbial activity and digestion in the rumen.

#### NH_3_ nitrogen

Supplementation treatments with DFM and Gambier did not show statistically significant differences (p > 0.05), although they tended to increase ruminal NH_3_ concentrations compared with the control. However, this increase must be balanced with nitrogen utilization efficiency by rumen microbes for optimal MPS. In this study, the ruminal NH_3_ concentrations ranged from 21.14 to 25.39 mg/100 mL. These values fall within the normal range reported by Leng [30], i.e., 1–34 mg/100 mL of rumen fluid and meet the optimal threshold for rumen microbial growth as stated by McDonald *et al*. [31], which is between 85 and 300 mg/L (equivalent to 8.5–30 mg/100 mL). NH_3_ concentration is closely associated with MPS, as rumen microbes use NH_3_ as a nitrogen source for protein synthesis. As shown in Table 5, NH_3_ values agreed with the MPS levels.

#### VFAs and A:P ratio

The addition of feed additives, particularly DFM and Gambier, significantly affected (p < 0.05) rumen fermentation profiles, including both VFA production and CH_4_ emission. Acetate was the most abundant VFA produced. Propionate is the primary precursor for gluconeogenesis, forming glucose, which is a major energy source for ruminants, while acetate and butyrate serve as precursors for long-chain FA synthesis [32]. The highest acetate production (82.68 mg/100 mL) was observed in P0. The decrease in acetate in treatments P3 and P5 may be attributed to an increased proportion of microbes favoring propionate-producing pathways. This shift, primarily triggered by the addition of DFM, occurs because yeast enhances metabolic pathways responsible for propionate synthesis by utilizing H_2_ under methanogenic conditions [33]. Soltan *et al*. [34] found that combining *Saccharomyces* with *Pichia manshurica* improved fermentation and increased propionate-producing bacteria.

Feeds with higher fermentable carbohydrates and probiotic inclusion tend to reduce the A:P ratio. In this study (Table 6), increasing DFM supplementation decreased the A:P ratio from 3.19 to 1.22, reflecting a higher propionate proportion. A reduced A:P ratio is associated with lower CH_4_ formation since acetate serves as a precursor for methanogenesis [35]. Here, A:P ratios ranged from 1.22 to 3.52, influencing energy metabolism efficiency. The higher A:P ratios observed in treatments P4 and P5 may reflect the influence of tannins in Gambier, which inhibit carbohydrate fermentation toward propionate and instead promote acetate production [36]. This increase in acetate could potentially lead to greater CH_4_ production, which is consistent with Alabi *et al*. [37].

#### Branched-chain VFAs

In this study, branched-chain VFA results differed significantly (p < 0.05). Feeds supplemented with Gambier showed a decreasing trend in branched-chain VFA production as the supplementation dose increased. The highest iso-butyrate (iC4) concentrations were found in P1 (2.16 mM) and P4 (2.03 mM), while the lowest were recorded in P3, P5, and P1 (1.21–1.31 mM). A similar pattern was observed for iC5, with the highest in P2 (3.05 mM) and the lowest in P3 and P5 (1.71–1.73 mM).

These findings suggest that DFM supplementation optimally supports ruminal protein fermentation, likely by increasing the activity of proteolytic bacteria that metabolize branched-chain amino acids. The elevated branched-chain VFA production in P2 aligns with the results of Polovyi *et al*. [38], who reported enhanced proteolytic enzyme activity in sheep supplemented with 1.2% EnzActive (Enzym Group, Ukraine). However, the significant decline in iC4 and iC5 production in P3 (1% DFM) indicates a possible inhibitory feedback effect at higher doses [39].

In Gambier-supplemented groups, the 1% dosage (P4) maintained high iC4 but not iC5 production, whereas the 2% dosage (P5) suppressed both, supporting the hypothesis that tannins act in a dose-responsive manner [40, 41]. High tannin levels can significantly inhibit microbial enzyme activity [42].

VFAs are produced through lipid and carbohydrate fermentation and serve as primary energy sources for ruminants. Increased total VFA indicates improved microbial fermentation efficiency. The high total VFA in P1 likely reflects the greater fermentable carbohydrate content supporting microbial growth. DFMs such as *Lactobacillus* or *Bacillus subtilis* shift fermentation toward propionate and suppress acetate formation [43], while tannins in Gambier inhibit acetogenic microbes, reducing acetate production [44].

#### CH_4_ gas production

The lowest CH_4_ production was observed in P4 (1% Gambier) and P5 (2% Gambier), demonstrating that Gambier supplementation effectively suppressed ruminal methanogenesis. This inhibition is primarily attributed to the high concentrations of condensed tannins (60.22%) and catechins (55.21%) in Gambier, which modulate rumen fermentation through multiple mechanisms: (1) Direct inhibition of methanogenic archaea [36, 42]; (2) protein–carbohydrate complex formation reducing H_2_ release [45]; and (3) redirection of metabolic H_2_ toward propionate synthesis via *Selenomonas ruminantium* activity [46, 47].

CH_4_ reduction reached 33.43% at 1% Gambier inclusion (P4) relative to the control, with no further improvement at 2% (P5). This indicates a threshold effect beyond which additional tannins offer limited benefit. Hassanat and Benchaar [44] similarly reported that moderate tannin levels reduced CH_4_ without further suppression at higher concentrations.

Comparable results have been observed by Acosta-Lozano *et al*. [48] and Adejoro *et al*. [49] with *Acacia mearnsii*, by Araiza-Ponce *et al*. [50] and Stifkens *et al*. [51] with *Leucaena leucocephala*, and by Rira *et al*. [52] with *Calliandra calothyrsus*, yielding CH_4_ reductions of 15%–35%. The higher reduction from Gambier may result from its synergistic catechin–tannin composition. However, *in vitro* conditions necessitate caution when extrapolating results; further *in vivo* validation is recommended.

DFM supplementation (P2: 0.5%; P3: 1%) also reduced CH_4_ moderately compared with the control but less effectively than Gambier. DFMs increase propionate-producing bacterial populations (*Propionibacterium*, *Megasphaera elsdenii*) that compete with methanogens for H_2_ [33, 53]. The lack of difference between 0.5% and 1% DFM suggests a dosage threshold associated with microbial colonization efficiency [54]. Strain adaptability to rumen conditions remains key for optimizing antimethanogenic effects.

#### MPS

In DFM-supplemented groups, MPS increased proportionally with DFM level: P2 (0.5%) yielded 146.06 mg/100 mL and P3 (1%) yielded 162.98 mg/100 mL. DFM enhances carbohydrate fermentation efficiency, supports microbial growth, and improves nitrogen utilization by stabilizing rumen activity [55]. *S. cerevisiae* reduces oxygen, stabilizes pH, and provides vitamins and enzymes that foster microbial synthesis [56]. Although increasing DFM from 0.5% to 1% improved MPS, the benefit plateaued, suggesting a colonization limit.

The highest MPS occurred in Gambier-supplemented treatments: P4 (1%) = 197.73 mg/100 mL; P5 (2%) = 207.70 mg/100 mL. This rise reflects tannins’ ability to form complexes with dietary protein, reducing degradation and increasing protein availability for microbial synthesis [57]. However, no statistical difference was observed between P4 and P5, indicating that 1% Gambier is the optimal inclusion rate.

#### FA profile

The main FAs produced included SFAs such as palmitic and stearic acids; MUFAs such as oleic acid; and PUFAs, including omega-3 and omega-6. Ruminants also produce trans-FAs (TFAs), such as trans-vaccenic acid and CLA, both of which have health benefits.

#### SFA

SFAs, myristic (C14:0), palmitic (C16:0), and stearic (C18:0), dominated, with total SFA = 70%–80%. Palmitic acid (C16:0) was the most abundant, highest in P3 (40.35%) and P4 (37.71%). These findings align with studies by Sears *et al*. [58] and Cozma *et al*. [59], which highlight palmitic and stearic acids as the primary ruminant lipids. Palmitic acid differences (p < 0.05) likely result from varying saturated fat protection against biohydrogenation. The palmitic contents of elephant grass and Gamal are 23% and 21.1%, respectively [60]. Rumen biohydrogenation converts unsaturated to SFAs via microbial hydrogenation, increasing SFA. According to Khattab *et al*. [61], this process neutralizes the toxic effects of unsaturated fats on microbes.

#### MUFA and PUFA

MUFAs and PUFAs varied, with PUFA = 7.16%–10.92%. PUFAs serve as CLA precursors but are prone to biohydrogenation. Essential FAs such as linoleic (C18:2 n-6) and α-linolenic (C18:3 n-3) are vital for health. The highest PUFA content occurred in P2 (10.92%), suggesting improved essential FA profiles. Given PUFAs’ anti-inflammatory roles, this may enhance meat nutritional quality.

#### CLA and TFAs

Ruminant lipid metabolism produced CLA and trans-isomers (trans-11, trans-15, cis-15 C18:1). CLA remained stable (1.7%–2.5%) and is recognized for anticarcinogenic and immunomodulatory functions [62]. Supplementation with fish oil or medium-chain FAs can further elevate beneficial FAs such as CLA [63]. Thus, manipulating dietary lipid sources could improve the nutritional and health-promoting value of ruminant-derived products.

### CONCLUSION

The results of this *in vitro* study demonstrated that supplementation with Gambier and DFM significantly influenced rumen fermentation characteristics, MPS, and CH_4_ mitigation without adversely affecting nutrient digestibility. DMD and OMD were improved in DFM-supplemented treatments (0.5% and 1%), indicating enhanced fiber degradation and microbial activity. The inclusion of Gambier reduced CH_4_ production by up to 33.43%, while also increasing NH_3_ concentration and MPS, particularly at 1% inclusion level. The A:P ratio decreased with DFM supplementation, reflecting a metabolic shift toward propionate formation, whereas Gambier treatments tended to favor acetate production due to tannin-mediated modulation of fermentation pathways. FA profiling revealed that DFM improved PUFA proportions, while Gambier maintained higher SFA content, reflecting different mechanisms of microbial regulation.

These findings have strong implications for sustainable ruminant production, particularly in tropical regions. Gambier, an abundant and low-cost local resource in Indonesia, exhibits considerable potential as a natural CH_4_ inhibitor due to its high condensed tannin and catechin contents. Its inclusion at moderate levels (around 1%) can effectively suppress methanogenesis while maintaining rumen fermentation efficiency. Meanwhile, DFM supplementation, especially at 0.5%, can enhance digestibility and fermentation stability, improving feed utilization and energy efficiency. The dual use of plant secondary metabolites and microbial additives represents a practical, environmentally friendly strategy to improve productivity and reduce GHG emissions in smallholder beef cattle systems.

The major strength of this study lies in its comparative design, which evaluated both microbial (DFM) and plant-based (Gambier) additives under controlled in vitro conditions using locally available substrates. The use of standardized methods for digestibility, fermentation, CH_4_ quantification, and FA profiling provides reliable, reproducible, and quantitative evidence for the effects of these feed additives. Furthermore, the study offers region-specific data relevant to tropical livestock systems that often rely on high-fiber diets and limited feed resources.

Despite these promising results, the findings are based on *in vitro* fermentation trials and may not fully replicate *in vivo* physiological and microbial interactions. The absence of dynamic rumen parameters such as passage rate, absorption, and animal performance indicators (e.g., feed intake, growth rate, or milk yield) limits the direct extrapolation of results to live animals. In addition, the long-term impacts of tannin-rich additives on nutrient absorption, feed intake, and animal health require further investigation to determine optimal inclusion levels that balance CH_4_ reduction with productivity.

Future studies should focus on *in vivo* validation to evaluate the long-term effects of Gambier and DFM supplementation on CH_4_ emissions, animal performance, and feed conversion efficiency. Advanced molecular approaches such as 16S rRNA sequencing and metagenomics should be employed to elucidate changes in the rumen microbiome and methanogenic communities in response to tannin and yeast supplementation. Investigating the potential synergistic effects of combining Gambier and DFM could further optimize CH_4_ mitigation and fermentation balance. Moreover, life-cycle assessment studies are recommended to quantify the overall reduction in GHG emissions and economic benefits at the farm level.

In conclusion, Gambier and DFM supplementation represent effective, eco-friendly, and locally sustainable strategies to improve rumen fermentation efficiency and reduce CH_4_ emissions in beef cattle feeding systems. The optimal inclusion rates identified in this study, 0.5% DFM and 1% Gambier, can serve as practical guidelines for formulating low-emission, high-efficiency ruminant diets. The integration of such additives supports the global goals of sustainable livestock production, aligning with the “One Health” concept by enhancing animal performance while mitigating environmental impact.

### AUTHORS’ CONTRIBUTIONS

BVU and MZ: Designed the study and drafted and reviewed the manuscript. EE and WN: Provided technical assistance and oversight. BVU, LSS, and ZI: Conducted the laboratory tests, performed the data analysis, and drafted the manuscript. All authors have reviewed and approved the final version of the manuscript.
